# Clinical and radiological characteristics of patients with pulmonary marginal zone lymphoma: A single center analysis

**DOI:** 10.1002/cam4.3096

**Published:** 2020-05-26

**Authors:** Muhammad Husnain, Russ Kuker, Isildinha M. Reis, Sunil Girish Iyer, Wei Zhao, Jennifer R. Chapman, Francisco Vega, Izidore S. Lossos, Juan Pablo Alderuccio

**Affiliations:** ^1^ Division of Hematology Department of Medicine University of Miami Miller School of Medicine Miami FL USA; ^2^ Division of Nuclear Medicine Department of Radiology University of Miami Miller School of Medicine Miami FL USA; ^3^ Department of Public Health Science University of Miami Miller School of Medicine Miami FL USA; ^4^ Biostatistics and Bioinformatics Core Resource University of Miami Miller School of Medicine Miami FL USA; ^5^ Division of Internal Medicine Department of Medicine University of Miami Miller School of Medicine Miami FL USA; ^6^ Division of Hematopathology Department of Pathology and Laboratory Medicine University of Miami Miller School of Medicine Miami FL USA; ^7^ Department of Molecular and Cellular Pharmacology Sylvester Comprehensive Cancer Center University of Miami Miller School of Medicine Miami FL USA; ^8^ Present address: MD Anderson Cancer Center Houston TX USA

**Keywords:** Pulmonary marginal zone lymphoma, radiographic findings, treatment outcomes

## Abstract

Pulmonary marginal zone lymphoma (PMZL) is the most common non‐Hodgkin lymphoma affecting the lung. PMZL is usually an indolent disease. Clinical and radiological variables associated with shorter survival are largely unknown and no consensus exists on preferred treatment strategy in PMZL. Herein we aimed to identify clinical and radiological features associated with shorter survival and inferior treatment outcomes. Forty patients with PMZL were analyzed. FDG‐avid disease was evident in most patients (93%) with staging PET/CT (n = 15). With a median follow‐up in treated patients (n = 38) of 8.4 years (range 0.07‐18.44), the median progression‐free survival (PFS) and overall survival (OS) were 7.5 years (95% CI 1.8‐9.5) and 15.7 years (95% CI 9.3‐NE) respectively. Shorter PFS was observed in patients who presented at diagnosis with elevated LDH, B symptoms, advanced stage and failed to achieve complete response (CR) after initial treatment. Patients with multifocal lung disease, extrapulmonary MZL and cavitary lesions on CT scans exhibited shorter PFS. Nevertheless, no clinical or radiologic findings were associated with shorter OS. All patients treated with surgery (n = 4) and radiation therapy (n = 3) achieved and remained in CR. No higher grade transformations occurred during the follow‐up period. PMZL exhibited excellent outcomes with a 15‐year PMZL‐related OS of 94.9% (95% CI: 81.25%‐98.7%). Radiation therapy and surgery are potentially curative strategies in localized PMZL.

## Graphical abstract

Elevated LDH, B symptoms, advanced stage and failure to achieve complete response after initial treatment are clinical variables associated with shorter progression‐free survival (PFS) in pulmonary marginal zone lymphoma (PMZL). Multifocal lung disease, extrapulmonary MZL and cavitary lesions on computed tomography scans have been identified as radiologic features associated with shorter PFS. Radiation therapy and surgery are potentially curative strategies in PMZL.

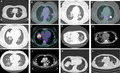

## 1 INTRODUCTION

Extranodal marginal zone B‐cell lymphoma of mucosa associated lymphoid tissue (MALT), also known as MALT lymphoma, comprises 6% to 8% of all non‐Hodgkin lymphomas (NHL).^1^ MALT lymphomas most commonly affect stomach; however, it has been reported in virtually all tissues.^2^, ^3^, ^4^ MALT lymphoma originating in the lung, also known as pulmonary marginal zone lymphoma (PMZL), is a rare disease representing 9%–14% of MALT lymphomas.^4^ However, it is the most common NHL affecting the lung. PMZL arises from bronchial associated lymphoid tissue (BALT) which is absent in normal healthy adults. Continuous antigen stimulation as occurs in association with inflammation, smoking, and autoimmune disorders (eg Sjögren's syndrome), leads to BALT appearance.^5^ This chronic antigenic stimulus is considered the underlying cause in PMZL.^6^ PMZL is associated with smoking in 35%–45% of patients^6^, ^7^, ^8^ and a possible association with *Achromobacter xylosoxidans* infection was described in Europe.^9^

Respiratory symptoms are usually present at the time of PMZL diagnosis and diverse patterns of lung abnormalities are observed on imaging studies.^10^ On computed tomography (CT) scans, PMZL usually manifests with bilateral (60%–70%) and multiple (70%–77%) lesions, without a clear topographic predominance.^10^ The most frequent patterns are lobar or segmental consolidation followed by nodules and masses.^6^, ^10^ The role of ^18^F‐deoxyglucose‐positron emission tomography/CT (FDG‐PET/CT) in MALT lymphoma is not well‐established. Several studies have shown FDG avidity in 80% of PMZL cases with an average SUV_max_ ranging from 3.3 to 7.5. It was suggested that PET/CT may detect previously unrecognized lesions during staging of PMZL.^10^, ^11^

Pulmonary marginal zone lymphoma carries a favorable outcome with a 5‐year overall survival (OS) rates of 90%.^10^, ^12^ Presence of mediastinal lymphadenopathy, extrapulmonary MZL, and use of chemotherapy regimens including anthracyclines or cyclophosphamide have been associated with shorter progression‐free survival (PFS) and time to progression.^6^, ^10^ The latter may reflect a more advanced disease needing systemic therapeutic approaches. Overall, clinical and radiological variables associated with worse outcome remain poorly understood.

Herein we report a single‐institution experience with a large cohort of patients with PMZL focusing on clinical features, radiological presentation, therapeutic approaches, and outcomes.

## 2 MATERIALS AND METHODS

All patients presenting with MALT lymphoma involving the lung and/or pleura diagnosed and treated at our institution between January 1995 and September 2019 were included in this analysis. Twenty‐seven patients presented with respiratory presentation and during staging were found to have extranodal MZL limited to the lungs. Additional 13 patients presented with respiratory complains with or without additional complains related to other organs and were found to have concomitant pulmonary and extrapulmonary extranodal MZL during staging. Patients with higher grade transformation (HGT) at presentation were excluded (n = 2) from this analysis. The patients were identified by a review of the Florida Cancer Registry database. The institutional review board approved this study, which followed the tenets of the Declaration of Helsinki. All specimens were reviewed by expert hematopathologists and MZL diagnosis was confirmed using the morphologic and immunophenotypic features defined by the WHO classification.^13^ Concomitant amyloid was detected at diagnosis in one patient only. Medical records were reviewed to obtain information on patients’ demographics, laboratory findings, smoking, staging, treatments, dates of diagnosis, relapse, transformation, and last follow‐up or death.

Staging evaluation was not standardized during the study interval, but included a complete physical examination; hematological and chemical survey with LDH; chest X‐rays and computerized tomography (CT) of the chest, abdomen, and pelvis in all the patients. PET‐CT, endoscopies, orbital or other magnetic resonance imaging (MRI), and ultrasound of thyroid or salivary glands were performed if clinically indicated. The decision to perform a staging bone marrow (BM) biopsy was left to the discretion of the treating oncologist. Based on the Ann Arbor staging classification,^14^ bilateral lung involvement was classified as stage IV. All the available chest X‐rays, CT scans, and FDG‐PET/CT scans were reviewed and revised by a board‐certified radiologist and nuclear medicine physician (RK).

### 2.1 Statistical analyses

Distributions of demographic and clinical characteristics were listed as frequency and percent. Missing values, except for BM involvement and presence of monoclonal gammopathy (MG), were grouped with the “low‐risk category” because after reviewing the clinical information they were felt more likely to be a negative/normal result. The MALT‐lymphoma International Prognostic Index (MALT‐IPI) was calculated as reported.^15^

Progression‐free survival was defined as the time from diagnosis to transformation after diagnosis, progression/relapse, or death, whichever occurred first. OS was defined as the time from diagnosis to death. Event‐free patients were censored at the date of last follow‐up. PFS and OS curves were estimated using the Kaplan‐Meier method and compared using the log‐rank test. Univariable and multivariable analyses using Cox proportional hazards regression were conducted to evaluate the effect of potential prognostic variables on PFS and OS. Multivariable models were derived using stepwise selection among candidate variables with cut‐offs *P* ≤ 50% to enter and *P* ≤ 5% to stay in the model. We first derived model 1 testing variables, except the MALT‐IPI. Next, we derived model 2, by forcing MALT‐IPI and testing other variables that were not part of that index.

## 3 RESULTS

### 3.1 Clinical characteristics of PMZL patients

Total of 40 patients with PMZL were diagnosed and treated at our institution between January 1995 and September 2019. Additional two patients with concomitant diagnosis of HGT were excluded from this analysis. Clinical characteristic of the 40 patients with PMZL are presented in Table 1. PMZL was diagnosed in 22 men (55%) and 18 women (45%) with a median age of 58.5 years (range 17.0‐80.0). At the time of diagnosis 67.5% had advanced disease (stage III‐IV), 22.5% presented with B symptoms, elevated LDH was detected in 32.5% of the patients and 37.5% presented with a MALT‐IPI score of 2. Staging BM biopsy was negative in 55% (22/30) of the patients with missing data in 10 patients. Five (12.5%) patients had an associated autoimmune disease but none had a history of Sjögren's syndrome. MG was present in 3 (7.5%) patients (IgM lambda, free light chain lambda, IgA kappa, and IgG lambda). Twelve patients (30%) had a history of smoking but data were missing for six (14%). No HGT was observed during the study follow‐up period.

**TABLE 1:** Characteristics of the study population

Variable	N	%
Total	40	100.0
Age		
<70	30	75.0
≥70	10	25.0
Gender		
Male	22	55.0
Female	18	45.0
Race		
White	15	37.5
Hispanic	18	45.0
Black	5	12.5
Other/Unknown	2	5.0
Tumor stage		
Stage I‐II	13	32.5
Stage III‐IV	27	67.5
LDH		
Normal LDH	27	67.5
Elevated LDH	13	32.5
Anemia (Hg < 12 g/dL)		
No	29	72.5
Yes	8	20.0
Unknown	3	7.5
Bone marrow involvement		
Negative	22	55.0
Positive	8	20.0
Unknown	10	25.0
Autoimmune disease		
No	35	87.5
Yes	5	12.5
Monoclonal gammopathy		
No	28	70.0
Yes	3	7.5
Unknown	9	22.5
B symptoms		
No B symptoms	31	77.5
B symptoms	9	22.5
PET/CT 5PS		
No PET/CT	25	62.5
DS 1‐3	1	2.5
DS 4‐5	14	35.0
Smoking status		
Non‐smoking	22	55.0
Smoking	12	30.0
Unknown	6	15.0
Organ involvement		
Extrapulmonary MZL	13	32.5
PMZL	27	67.5
Mediastinal lymphadenopathy (ML)		
Not present	16	40.0
Present	18	45.0
Unknown	6	15.0
Pleural effusion (PE)		
Not present	27	67.5
present	7	17.5
Unknown	6	15.0
Cavitation		
Not present	31	77.5
Present	3	7.5
Unknown	6	15.0
Mass		
Not present	19	47.5
Present	15	37.5
Unknown	6	15.0
Size		
<2 cm	4	10.0
2‐5 cm	18	45.0
>5 cm	9	22.5
0/Unknown	9	22.5
Unifocal/Multifocal		
Unifocal	12	30.0
Multifocal	22	55.0
Unknown	6	15.0
MALT‐IPI score		
0	9	22.5
1	14	35.0
2	15	37.5
3	2	5.0

Abbreviations: 5PS, five‐point scale; DS, Deauville score; Hg, hemoglobin; LDH: lactate dehydrogenase; MALT‐IPI, mucosa‐associated lymphoid tissue lymphoma international prognostic index; MZL, marginal zone lymphoma; PET/CT, positron emission tomography; PMZL, pulmonary marginal zone lymphoma.

### 3.2 Pulmonary radiological characteristics of PMZL patients

The involvement was unilateral in 29 (72%) and bilateral in 11 (28%) patients. Lung involvement was multifocal in 22 (55%) of patients. Twenty‐seven patients had lung only PMZL while 13 patients had extrapulmonary MZL with lung involvement. From 27 patients with only lung involvement, nine (33.3%) had stage IV due to multiple lung lesions, five (18.5%) due to BM involvement, while 13 (48.2%) had stage I disease.

**FIGURE 1:**
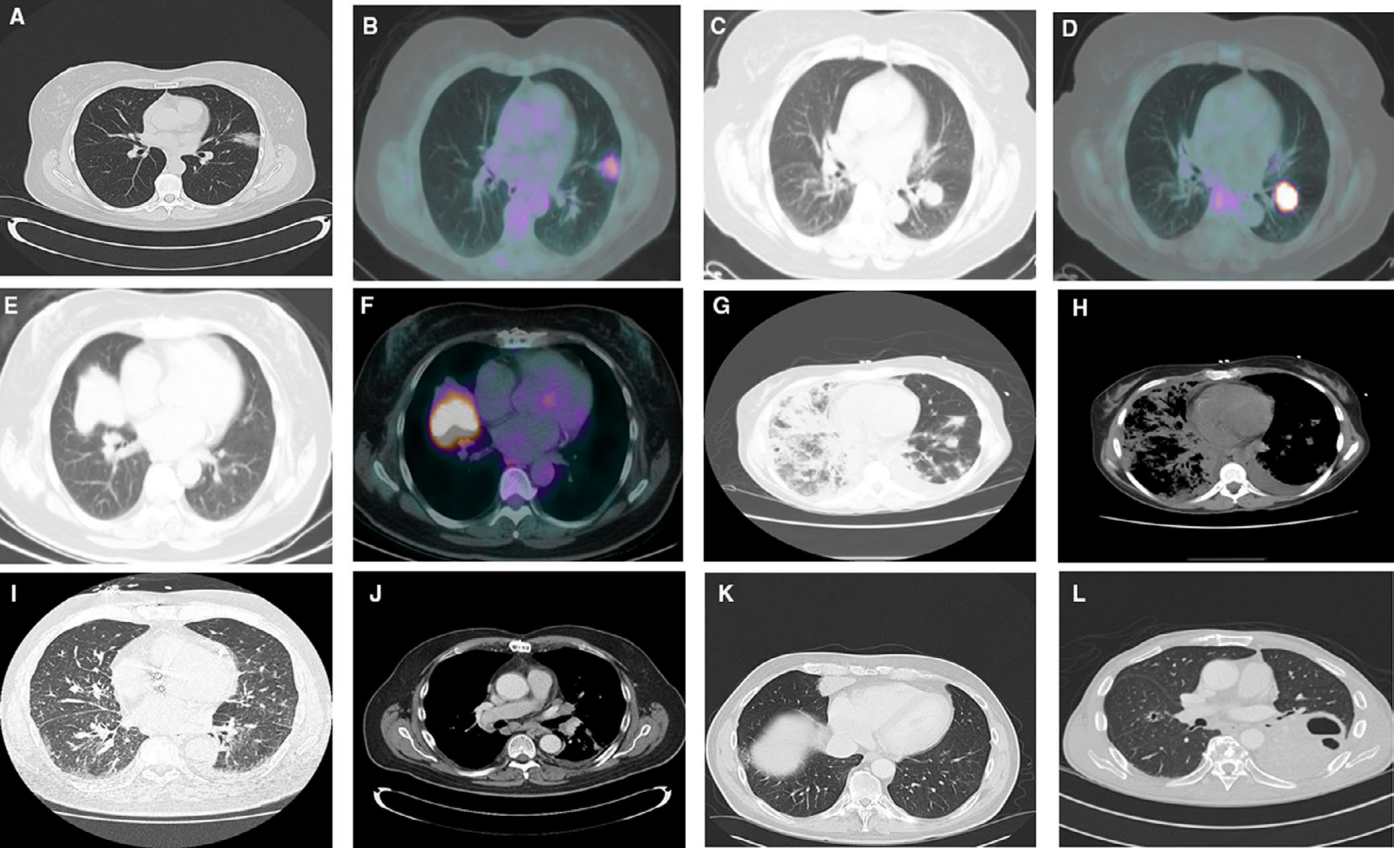
Radiological manifestations of pulmonary marginal zone lymphoma. A and B, focal ground glass opacity with air bronchograms and mild FDG‐uptake in the left upper lobe; (C and D), nodular lesion with intense FDG‐uptake in the left lower lobe; (E and F), pulmonary mass with intense FDG‐uptake in the right upper lobe; (G and H), multifocal bilateral lung parenchymal opacities with small left pleural effusion; (I and J), nodular interstitial thickening indicating lymphangitic spread of lymphoma, also noted to have mediastinal and hilar adenopathy on CT with contrast; (K), focal area of consolidation with homogeneous enhancement in the right middle lobe on CT with contrast; (L), cavitary consolidation in the left lower lobe with an additional cavitary nodule in the right middle lobe

Radiological scans were available and were reviewed for 34 patients and shown in Table 1 and Figure 1. Consolidation or air‐bronchogram pattern were the most common presenting findings seen on CT scans in 22 (65%) of patients, followed by lung nodules in 18 (53%) patients. Ground glass opacities (GGO) were seen in 14 (41%), while pleural effusion was observed in seven (21%) patients. Mass like lesion was detected in 15 (44%) patients, cavitary lesions in three (9%), and lymphangitic spread was seen on chest CT in three (9%) patients. We measured lesion size and classified patients based on the size of their largest lesion into three categories: <2 cm in 4 (12%) patients, 2‐5 cm in 18 (53%) and more than 5 cm in nine (27%), while lesion size data were unavailable for three (9%) patients.

FDG‐PET/CT scan data were available in 15 patients, with 14 (93.3%) patients having FDG‐avid disease, as demonstrated by presence of hypermetabolic lesions corresponding to the areas of PMZL. Fourteen patients demonstrated a Deauville score of 4‐5, one patient demonstrated a Deauville score of 1 (Table 1). The patient with low FDG‐avidity presented with ground‐glass opacities, air‐bronchogram, and nodular lesions of 2‐5 cm on CT scans. SUV max in FDG avid cases ranged from 2.5 to 9.1 with a median SUV max of 4.0. No patient was upstaged based on the PET/CT scan.

### 3.3 Treatment

Initial treatment at the time of PMZL diagnosis in our cohort of 40 patients consisted of immunotherapy, chemotherapy or both in 26 (65%) patients, radiation therapy only in three (7.5%) patients, combined modality therapy (radiation and immuno/chemotherapy or immuno/chemotherapy and surgery) in five (12.5%) patients, and surgical resection in four (10%) patients (Table 2). Two (5%) of the 40 patients were never treated and remained on active surveillance. The overall response rate in 38 treated patients was 86.9% (25 CR and 8 PR). Overall response per treatment modality was 80.7% (CR 15 and PR 6) for immuno/chemotherapy only, 100% (CR 3) for radiation therapy only, 100% (CR 3 and PR 2) for combined modality, and 100% (CR 4) for surgical resection. It is important to note that all the patients treated with surgical resection, radiation therapy, or combined modality therapy had localized disease only.

**TABLE 2:** Treatments and response

Variable	N	%
Total	40	100.0
Treatment		
Active surveillance	2	5.0
Surgery only	4	10.0
RT only	3	7.5
Chemo only	26	65.0
Chemo + surgery	1	2.5
Chemo + RT	4	10.0
Chemotherapy regimens	31	100
Rituximab	10	32.2
R‐CVP	6	19.3
R‐CHOP	5	16.1
Ibritumomab Tiuxetan/rituximab	5	16.1
Bendamustine/Rituximab	2	6.4
Rituximab/Lenalidomide	1	3.3
Rituximab/Chlorambucil	1	3.3
Other^a^	1	3.3
Clinical response (n = 38 excluded 2 untreated)		
CR	25	65.8
PR	8	21.1
SD	1	2.6
PD	4	10.5

Abbreviations: CR, complete response; PD, progression of disease; PR, partial response; R‐CHOP: rituximab, cyclophosphamide, doxorubicin, vincristine and prednisone; R‐CVP, rituximab, cyclophosphamide, vincristine and prednisone; RT, radiation therapy; SD, stable disease.

^a^ Rituximab/Methotrexate/Procarbazine.

### 3.4 Progression‐free survival and overall survival

With a median follow‐up of 8.4 years (range 0.07‐18.44), 22 PFS events (17 relapses and 5 deaths) were seen among the 38 treated patients. All the PFS events were seen among the 33 patients treated with immuno/chemotherapy or combined modality therapy while there were no PFS events in patients treated with surgery or radiation therapy alone. Table 3 summarizes therapies given to patients upon relapse and patients’ outcome. The seven patients treated with either surgery (n = 4) or radiation therapy (n = 3), as primary modalities, remained alive and free of disease. Two patients on observation also remained alive and stable, not requiring any further therapy after 8.4 and 8.6 years of follow‐up respectively.

**TABLE 3:** Detailed characteristics of 17 relapsed PMZL patients

Patient	Site of disease	Initial treatment	Response 1	Site of relapse	Treatment	Response 2	Status	Cause of death
1	Lung, pleural effusion	Rituximab	CR	Lung, Bone, BM	R‐CVP	CR	Alive	NA
2	Lung, pleural effusion	Rituximab	CR	Lacrimal gland, lung, LNs	BR	SD	Alive	NA
3	Lung, gastric, skin	Ibritumomab Tiuxetan/rituximab	CR	Skin, subcutaneous soft tissue with skin amyloidosis	R‐CVP	SD	Deceased	Unknown
4	Lung, LNs, adrenal, Horner syndrome	Rituximab	PD	Lung, LNs, adrenal, Horner syndrome	R‐CHOP, HDMTX	CR	Alive	NA
5	Lung	R‐CHOP	CR	Lung, LNs	BR	CR	Alive	NA
6	Pleural effusion, LNs	Rituximab	PD	Pleural effusion	BR	PR	Alive	NA
7	Lung, spinal cord, Bone, +CSF	Rituximab, procarbazine, IT‐MTX‐AraC	PD	New brain lesion	DHAP, HDMTX and IT‐MTX, AraC	CR	Alive	NA
8	Lung, spleen, bone	Ibritumomab Tiuxetan/rituximab	PR	Lung	RCVP	PR	Alive	NA
9	Lung	Rituximab	CR	Lung	BR, RT	CR	Deceased	Unknown
10	Lung and BM	Rituximab + Surgery	CR	Parotid	Rituximab, RT	CR	Deceased	Unknown
11	Lung and BM	Rituximab	CR	Lung	BR	CR	Deceased	Unknown
12	Lung, pelvic mass	Rituximab	CR	Lung, Gastric, perineal	Rituximab	PR	Alive	NA
13	Lung, Renal, LNs	R‐CVP	PR	Lung, Renal	Ibritumomab Tiuxetan/rituximab	PR	Alive	NA
14	Lung, LNs	R‐CVP	CR	Lung	R‐CHOP	PR	Alive	NA
15	Lung, gastric, BM	R‐CVP	PD	Gastric and lung	Surveillance	SD	Deceased	SCC of head and neck
16	Lung	R‐CHOP	PR	Lung	Rituximab Chlorambucil, Gamma knife	CR	Alive	NA
17	Lung, LNs	BR	CR	LNs	Surveillance	SD	Alive	NA

Boldface indicates significance at P < .05. Abbreviations: BM, Bone marrow; BR, bendamustine, rituximab; CR, complete response; DHAP, dexamethasone, cytarabine, cisplatin; HDMTX, high dose methotrexate; IT MTX, intrathecal methotrexate; LNs, lymph nodes; NA, not applicable; PD, disease progression; PMZL, pulmonary marginal zone lymphoma; PR, partial response; R‐CHOP, rituximab, cyclophosphamide, doxorubicin, vincristine, prednisone; R‐CVP, rituximab, cyclophosphamide, vincristine, prednisone; SCC, squamous cell carcinoma; SD, stable disease.

There were 10 deaths during the follow‐up period, including two attributed to PMZL (n = 1) or PMZL treatment (n = 1). A patient with the shortest follow‐up of 1.5 months died of PMZL. Four patients died secondary to other causes: squamous cell carcinoma of head and neck (n = 1), peripheral T cell lymphoma that developed after renal transplant (n = 1), autoimmune hemolytic anemia (n = 1), and stab wound to the chest (n = 1). The cause of death was unknown in four patients who died in CR (n = 3) or with SD (n = 1) at the time of last follow‐up.

**FIGURE 2:**
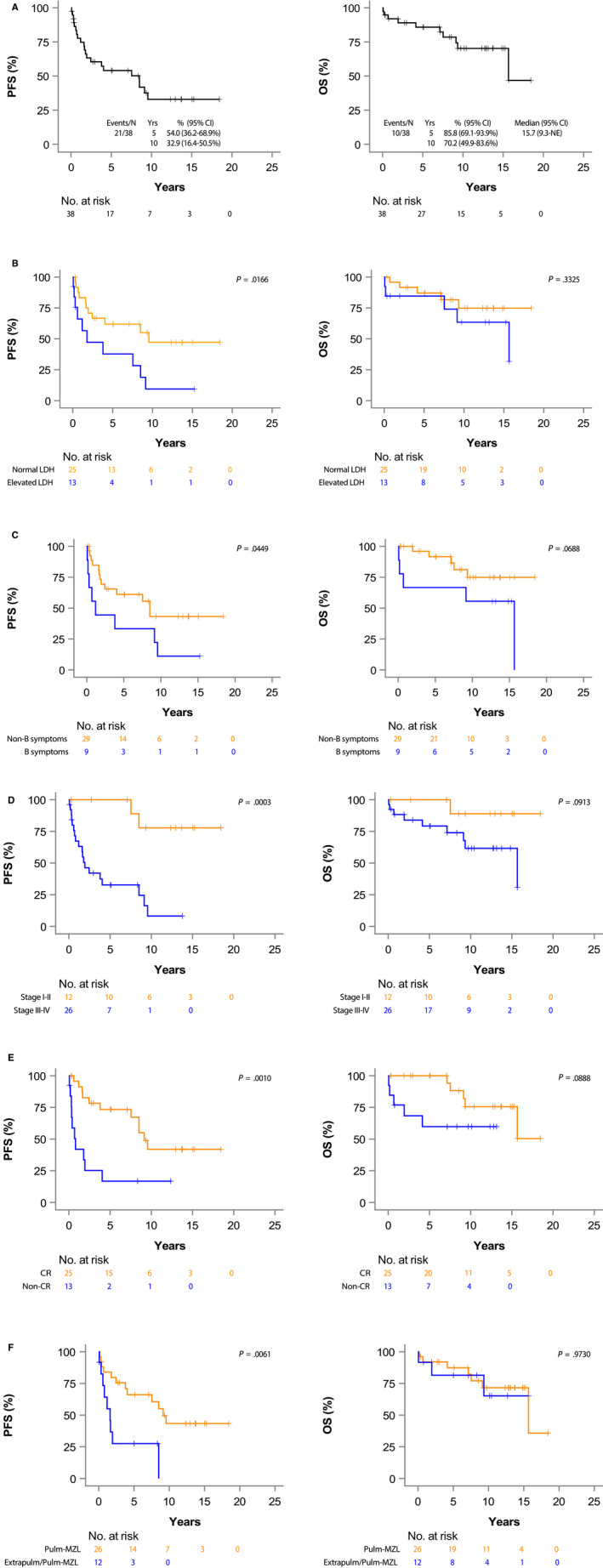
Kaplan‐Meier curves for progression free survival (PFS) and overall survival (OS) in 40 treated patients with pulmonary marginal zone lymphoma based on clinical features present at diagnosis. A, in all the patients; (B), by lactate dehydrogenase (LDH); (C), by B symptoms; (D), by stage (I‐II vs III‐IV); (E), by complete response (CR) after initial therapy; (F), by presence of extrapulmonary (Extrapulm) marginal zone lymphoma (MZL)

In treated patients, the median PFS was 7.5 years (95% CI 1.8‐9.5) and the median OS was 15.7 years (95% CI 9.3‐NE; Figure 2A). The 10‐year PFS and OS were 32.9% (95% CI 16.4%‐50.5%) and 70.2% (95% CI 49.9%‐83.6%) respectively.

In the nine patients who initially were treated with rituximab alone, the mPFS was 7.5 (1.3‐9.1) years and in patients achieving CR, the median duration of response was 5.2 years (range 0.6‐9.5).

We then analyzed prognostic factors associated with PFS and OS (Table 4). PFS was significantly shorter in patients with elevated LDH at presentation (Figure 2B), presence of B symptoms (Figure 2C), advanced stage (Figure 2D), failure to achieve CR after initial treatment (Figure 2E), and presence of extrapulmonary MZL (Figure 2F). Nevertheless, none of these variables affected the OS (Figure 2B‐F). Univariable analyzes (Table 4) showed association between shorter PFS and elevated LDH at presentation (HR = 2.57, 95% CI 1.16‐6.51, *P* = .022), advanced stage (HR = 9.84, 95% CI of 2.24‐43.3, *P* = .002), and failure to achieve CR after initial treatment (HR = 3.95, 95% CI 1.64‐9.52, *P* = .002). Patients without extrapulmonary MZL had longer PFS (HR = 3.41, 95% CI 1.35‐8.60, *P = *.009). However, none of these variables were associated with shorter OS (Table 4).

**TABLE 4:** Univaria Cox models for progression‐free survival (PFS) and overall survival (OS) in 38 treated patients

Variable	N	PFS (21 events)					OS (10 events)				
		Events		HR (95% CI)		*P*	Death		HR (95% CI)		*P*
Gender											
Male	20	13	Reference				5	Reference			
Female	18	8	0.63 (0.26, 1.51)		.299		5	1.11 (0.32, 3.88)		.872	
Race											
White	14	9	Reference				4	Reference			
Hispanic	17	7	0.75 (0.28, 2.04)		.574		4	1.30 (0.32, 5.31)		.711	
Black	5	5	2.58 (0.85, 7.82)		.095		2	1.69 (0.30, 9.51)		.553	
Other/Unknown	2	‐	NE				‐	NE			
Age											
<70	30	15	Reference				6	Reference			
≥70	8	6	2.11 (0.81, 5.48)		.126		4	2.59 (0.72, 9.28)		.144	
Tumor stage											
Stage I‐II	12	2	Reference				1	Reference			
Stage III‐IV	26	19	9.84 (2.24, 43.3)		**.002**		9	4.98 (0.63, 39.3)		.128	
LDH											
Normal LDH	25	11	Reference				5	Reference			
Elevated LDH	13	10	2.75 (1.16, 6.51)		**.022**		5	1.85 (0.52, 6.58)		.339	
MALT‐IPI											
0‐1	22	8	Reference				4	Reference			
2‐3	16	13	3.98 (1.63, 9.73)		**.002**		6	2.02 (0.56, 7.27)		.280	
B symptoms											
Non‐B symptoms	29	13	Reference				5	Reference			
B symptoms	9	8	2.41 (0.99, 5.84)		.052		5	3.02 (0.87, 10.5)		.083	
Smoking status											
Non‐smoking	21	14	Reference				8	Reference			
Smoking	11	4	0.46 (0.15, 1.41)		.175		‐	NE			
Unknown	6	3	0.51 (0.14, 1.78)		.288		2	0.81 (0.17, 3.90)		.791	
Lung disease											
Pulm MZL	26	12	Reference				3	Reference			
Pulm/Extrapulm–MZL	12	9	3.41 (1.35, 8.60)		**.009**		7	1.02 (0.26, 3.97)		.973	
Mediastinal lymphadenopathy (ML)											
Non‐ML	14	5	Reference				2	Reference			
ML	18	12	2.72 (0.94, 7.86)		.065		6	3.28 (0.64, 16.9)		.155	
Unknown	6	4	1.41 (0.38, 5.27)		.610		2	1.27 (0.17, 9.67)		.815	
Clinical response											
CR	25	11	Reference				5	Reference			
Non‐CR	13	10	3.95 (1.64, 9.52)		**.002**		5	2.97 (0.80, 11.1)		.105	
Pleural effusion (PE)											
Non‐PE	25	12	Reference				6	Reference			
PE	7	5	2.28 (0.80, 6.49)		.124		2	1.52 (0.30, 7.65)		.608	
Unknown	6	4	0.94 (0.30, 2.93)		.916		2	0.72 (0.13, 3.89)		.707	
Cavitation											
Non‐Cavitation	29	15	Reference				7	Reference			
Cavitation	3	2	49.2 (4.18, 579.00)		**.002**		1	2.20 (0.27, 18.1)		.462	
Unknown	6	4	0.86 (0.28, 2.60)		.787		2	0.71 (0.14, 3.75)		.690	
Mass											
Non‐Mass	18	9	Reference				7	Reference			
Mass	14	8	1.07 (0.41, 2.79)		.883		1	0.16 (0.02, 1.31)		.088	
Unknown	6	4	0.81 (0.25, 2.66)		.734		2	0.43 (0.08, 2.14)		.300	
Tumor size (cm)											
<2 cm	4	2	Reference				1	Reference			
2‐5 cm	16	7	0.83 (0.17, 4.00)		.813		4	1.45 (0.13, 16.6)		.767	
>5 cm	9	7	1.94 (0.40, 9.37)		.411		3	2.20 (0.18, 26.9)		.537	
0/Unknown	9	5	0.61 (0.12, 3.16)		.555		2	0.64 (0.06, 7.32)		.719	
Unifocal/Multifocal											
Unifocal	11	2	Reference				‐	Reference			
Multifocal	21	15	7.61 (1.72, 33.7)		**.007**		8	NE			
Unknown	6	4	3.30 (0.60, 18.1)		.168		2	NE			

Boldface indicates significance at *P* < .05. Abbreviations: MZL, marginal zone lymphoma; NE, not estimable; Pulm, pulmonary; extrapulm; extrapulmonary.

A multivariable analysis (Table 5) demonstrated that age ≥70 (HR = 3.22, 95% CI 1.04‐9.95, *P* = .042), elevated LDH (HR = 3.55, 95% CI 1.38‐9.14, *P* = .009), advanced stage (HR = 6.76, 95% CI 1.48‐31.0, *P* = .014), not achieving CR (HR = 7.18, 95% CI 2.25‐22.9, *P* = .001), and MALT‐IPI of 2‐3 (HR of 7.24, 95% CI 2.55‐20.53, *P* = .001) were associated with shorter PFS. However, none of these variables were associated with shorter OS.

**TABLE 5:** Multivariable Cox models for progression‐free survival (PFS) and overall survival (OS) in 38 treated patients

Variable	Category	PFS (21 events)		OS, event (10 events)	
		HR (95% CI)	*P*	HR (95% CI)	*P*
Model 1					
Age	<70	Reference		Reference	
	≥70	3.22 (1.04, 9.95)	**.042**	3.25 (0.76, 13.9)	.111
Tumor stage	Stage I‐II	Reference		Reference	
	Stage III‐IV	6.76 (1.48, 31.0)	**.014**	2.42 (0.27, 21.7)	.429
LDH	Normal LDH	Reference		Reference	
	Elevated LDH	3.55 (1.38, 9.14)	**.009**	1.68 (0.44, 6.40)	.450
Clinical response	CR	Reference		Reference	
	Non‐CR	7.18 (2.25, 22.9)	**<.001**	3.39 (0.76, 15.2)	.110
Model 2					
MALT‐IPI	0‐1	Reference		Reference	
	2‐3	7.24 (2.55, 20.53)	**<.001**	2.09 (0.58, 7.54)	.260
Lung disease	Pulm‐MZL	Reference		Reference	
	Extrapulm/Pulm‐MZL	2.49 (0.99, 6.25)	.052	0.84 (0.21, 3.30)	.803
Clinical response	CR	Reference		Reference	
	Non‐CR	7.33 (2.58, 20.85)	**<.001**	3.03 (0.81, 11.32)	.099

Boldface indicates significance at *P* < .05. Abbreviations: CR, complete response; LDH, lactate dehydrogenase; MALT‐IPI, mucosa‐associated lymphoid tissue lymphoma international prognostic index; MZL, marginal zone lymphoma; Pulm, pulmonary; extrapulm; extrapulmonary.

**FIGURE 3:**
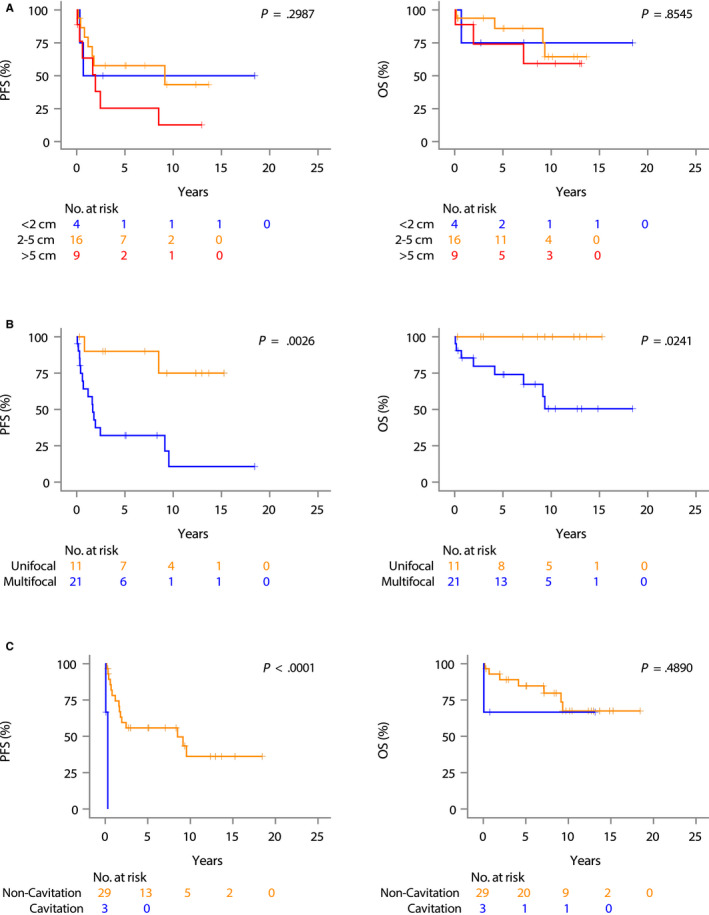
Kaplan‐Meier curves for progression free survival (PFS) and overall survival (OS) in 34 treated patients with pulmonary marginal zone lymphoma based on radiological findings at diagnosis. A, by size of lesion (<2 cm vs 2‐5 cm vs >5 cm); (B), by site of disease (unifocal vs multifocal disease); (C), by presence of cavitation

There were no differences in PFS and OS by lesion size (Figure 3A). Patients with multifocal disease on CT chest tended to have shorter PFS as compared to patients with unifocal disease (HR = 7.61, 95% CI of 1.72‐33.7, *P* = .007; Figure 3B), as also did patients with cavitary lesions (HR = 49.2, 95% CI 4.18‐579.00, *P* = .002; Figure 3C). However, radiologic findings were not associated with statistically significant shorter OS. (Figure 3A,C).

## 4 DISCUSSION

Pulmonary marginal zone lymphoma is a rare lymphoma, usually seen in patients older than 60 years old and characterized by an indolent course.^8^ The present study aimed to identify clinical and radiological findings associated with shorter survival in a large cohort of patients from a single institution followed for more than 8 years. The overall outcome was excellent with only 10 deaths, most of which were non‐PMZL related.

In line with prior studies, most patients included in our cohort presented with normal LDH (66.7%), absence of B symptoms (78.6%), and negative staging BM biopsy (57.1%); however, advanced stage (66.7%) at diagnosis was more frequent in our cohort compared to prior studies.^8^, ^10^ Radiologically, consolidation and nodule(s) followed by pulmonary mass were the most common radiological findings, as previously reported^6^, ^10^; however, we also observed lymphangitic spread and cavitations that are not commonly reported.^5^, ^8^, ^10^

Clinical factors associated with shorter survival in PMZL are still largely unknown and controversial. Oh et al reported shorter median time to progression and 5‐year OS in PMZL patients presenting with extrapulmonary (1.1 vs 6.2 years; *P* = .030 and 65.5% vs 95.95; *P* = .016, respectively) and lymph node involvement (2.4 vs 5.6 years, *P* = .024 and 80.7% vs 94.7%; *P* = .025 respectively).^6^ However, extrapulmonary MZL was not associated with inferior prognosis in another study.^10^ In our study, extrapulmonary MZL was associated with shorter PFS. Additional clinical factors associated with shorter PFS in the current study were advanced stage, elevated LDH, failure to achieved CR after frontline therapy, presence of cavitary lesions, and multifocal disease. In multivariable analysis, MALT‐IPI>1 and independent variables comprising this prognostic score as well as inability to achieve CR after frontline treatment were associated with shorter PFS. However, we did not identify any clinical or radiological finding associated with shorter OS likely due to the fact that majority of causes of death in our patients were unrelated to lymphoma with only two patients succumbing to lymphoma. These findings indicate excellent prognosis PMZL patients with lymphoma‐related OS of 94.9% (95% CI 81.25%‐98.7%) at 5, 10, and 15 years respectively.

Recent studies have demonstrated the utility of FDG‐PET/CT in small cohorts of PMZL with FDG‐avidity in 80%‐100% of patients.^11^ Our study supports prior observations demonstrating FDG‐avidity in PMZL with most patients presenting FDG‐uptake above liver background. Only one patient had disease detected on PET/CT that was not previously seen on CT scans, but not leading to upstaging. Furthermore, PET/CT was not diagnostic for BM involvement in our patients, however, the number of analyzed cases is small.

Higher grade transformation, reported in 3.8%‐8% of patients with MZL,^16^, ^17^, ^18^ is typically associated with shorter survival—a 5‐year OS of 65% after HGT.^17^ The incidence of HGT in PMZL patients is largely unknown. In a large study (n = 467) evaluating clinicopathological features of HGT in MALT lymphoma, none of the patients with PMZL transformed to DLBCL.^18^ Similarly, none of our patients experienced HGT during follow‐up. This finding in combination with low lymphoma‐associated mortality may suggest a different biology and natural history of PMZL in comparison to MALT lymphoma originating in other locations.

Currently, there is no standard/uniform treatment approach for PMZL patients. This is due to absence of large studies and randomized clinical trials, as well as favorable outcomes with multiple therapeutic modalities.^8^ Accepted treatment options include observation, radiation, surgical resection, and immunochemotherapy. Leyfman et al reported a 6‐year event‐free survival (EFS) and OS of 63% (95% CI 50%‐80%) and 88% (95% CI 77%‐100%), respectively, in patients managed with active surveillance.^19^ Nevertheless, complete surgical resection have resulted in excellent long‐term disease free survival and may be curative in localized PMZL patients.^20^, ^21^ Our findings support these prior observations, as all our patients treated with surgery achieved CR and remained without evidence of disease relapse after a prolonged follow‐up. Similarly, patients treated with radiation therapy (median dose 34 Gy, range 30‐36) achieved excellent outcomes providing further evidence that these two modalities may be preferentially considered for treatment of patients with localized PMZL.

Immunochemotherapy is usually reserved for symptomatic patients with extensive disease. Okamura et al evaluated the activity of a single agent rituximab in PMZL (n = 8) that resulted in an ORR of 75% (CR 62.5% and PR 12.5%) and mPFS of 5.5 years (range 0.8‐7.26 years).^22^ In our patients treated with rituximab as a single agent, the ORR was 89% and mPFS was 7.5 years (range 1.3‐9.1 years), representing an excellent option in patients requiring systemic therapy.

In summary, our findings confirm excellent outcomes and possible cure in patients with localized PMZL treated with surgery or radiation therapy. Several clinical and radiological features associated with shorter PFS were identified. However, no clinical characteristics associated with shorter OS were detected, likely due to the indolent nature and low PMZL‐related mortality in these patients. Multicenter collaborative studies are needed to define the best therapeutic approach in PMZL patients.

## CONFLICT OF INTEREST

ISL has served on advisory boards from Seattle Genetics, Janssen Scientific and Verastem. JPA has received honoraria from Targeted Oncology, OncLive and Oncinfo, and immediate family member has served on advisory boards from Puma Biotechnology, Inovio Pharmaceuticals, Agios Pharmaceuticals, Forma Therapeutics and Foundation Medicine.

## AUTHOR CONTRIBUTIONS

MH: collected and analyzed the data, and wrote the manuscript; RK: performed review of images; SGI: collected the data; WZ and IMR: analyzed the data and wrote the manuscript; JRC and FV: performed review of diagnostic biopsies and confirmed diagnosis; ISL: conceptualized and designed the study, was involved in the treatment of these patients, analyzed the data, and wrote the manuscript; JPA: collected the data, conceptualized and designed the study, analyzed the data, and wrote the manuscript. All authors read and approved the final version of the manuscript.

## Data Availability

All the inquiries regarding data will be provided by the corresponding author.
